# Long‐term ketogenic diet therapy improves mitochondrial encephalopathy with lactic acidosis and stroke‐like episodes (MELAS): A case report

**DOI:** 10.1111/cns.14274

**Published:** 2023-05-19

**Authors:** Fang He, Lingqi Ye, Pu Miao, Jiong Zhou, Yao Ding, Shuang Wang

**Affiliations:** ^1^ Department of Nutrition, Second Affiliated Hospital, School of Medicine Zhejiang University Hangzhou China; ^2^ Department of Neurology, Second Affiliated Hospital, School of Medicine Zhejiang University Hangzhou China; ^3^ Department of Pediatrics, Second Affiliated Hospital, School of Medicine Zhejiang University Hangzhou China

**Keywords:** epileptic seizures, ketogenic diet, MELAS syndrome

Dear Editor,

A ketogenic diet (KD) produces a broad‐spectrum of effects in drug‐resistant epilepsy. Several case studies suggest KD may be beneficial for alleviating muscular symptoms as well as seizure control in some patients with mitochondrial diseases.^1^, ^2^, ^3^ Notably, the efficacy of KD therapy may produce different results for different types of mitochondrial diseases. In a recent study, half of children with mitochondrial diseases had >50% seizure reduction on a 3‐month KD therapy, and 90% of those with mitochondrial encephalopathy with lactic acidosis, and stroke‐like episodes (MELAS) syndrome responded to the treatment.^4^ At present, the outcome of long‐term KD therapy is not well known in mitochondrial diseases, including MELAS syndrome. Herein, we described the long‐term effects of KD on a female adult with MELAS syndrome. Besides seizure control, KD alleviated many other symptoms and delayed the occurrence of stroke‐like episodes for over 3.5 years.

## CASE PRESENTATION

**FIGURE 1:**
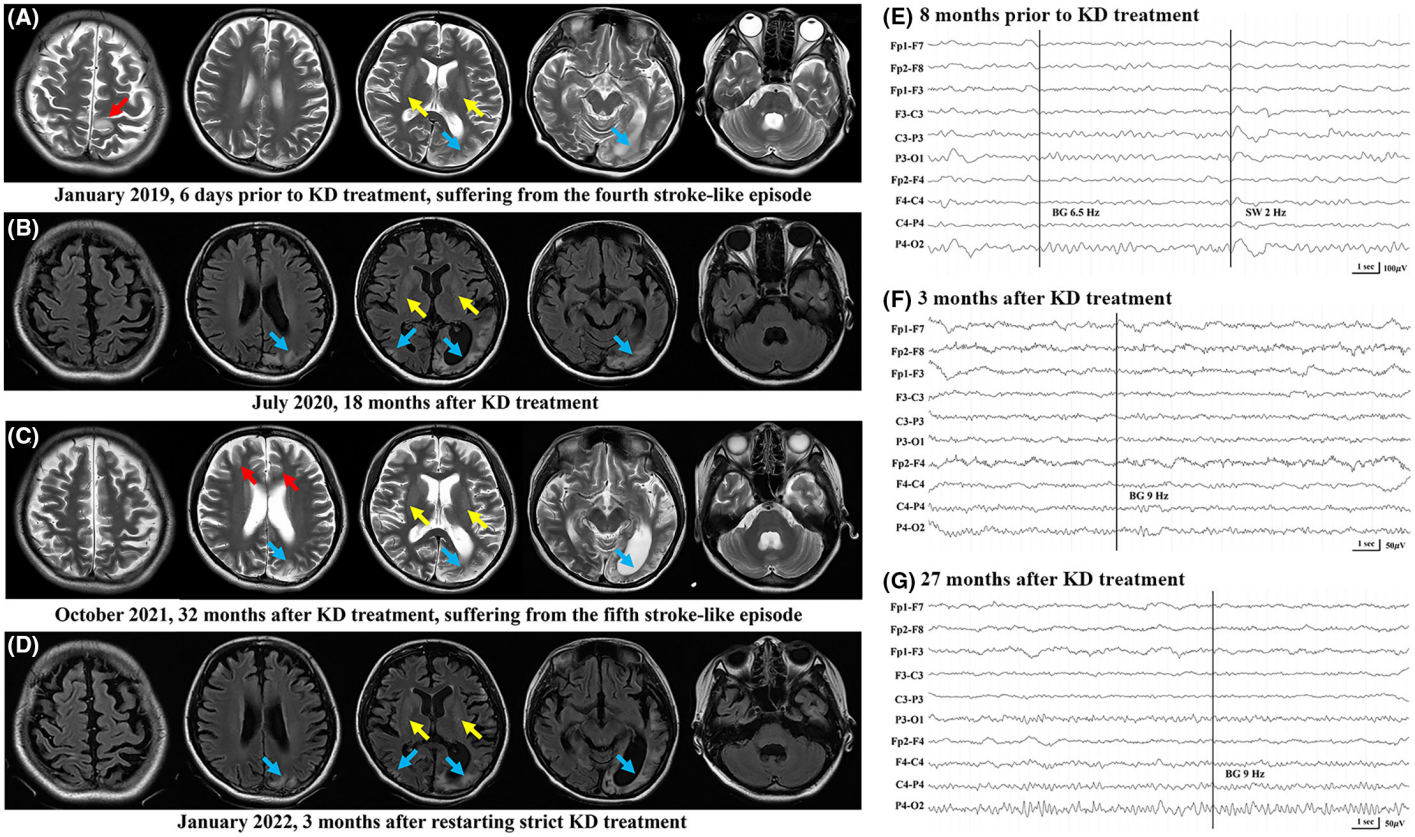
Chronological MRI findings and EEG examinations. (A) Six days prior to KD treatment, MRI (T2 images) showed a new lesion in the right parietal lobe, obsolete lesions in the bilateral posterior quadrants (more severe on the left) and mild cortical atrophy and bilateral basal ganglia calcification. (B) Eighteen months after KD treatment, T2‐FLAIR images showed no apparent change of the cortical volume. (C) On her fifth stroke‐like episode caused by non‐compliance with KD treatment, T2 images showed new lesions in the bilateral frontal lobes and the right parietal lobe were detected. (D) Three months after restarting strict KD treatment, the new responsible lesions resolved (T2‐FLAIR images). (A–D) Bilateral basal ganglia calcification (yellow arrows) was consistently observed. New lesions, red arrows; obsolete lesions, blue arrows. (E) Eight months prior to KD treatment, EEG recording showed a posterior background of 6–7 Hz and intermittent diffused slow activity of 2–4 Hz, which suggested a moderate encephalopathy. (F) Three months after KD treatment the posterior background was normalized (8–9 Hz) and maintained thereafter (G). Meanwhile, diffuse slowing and a normal posterior background suggested a mild encephalopathy. EEG, electroencephalogram; FLAIR, fluid attenuated inversion recovery; KD, ketogenic diet; MRI, magnetic resonance imaging.

A 20‐year‐old girl with uneventful family history and normal early developmental milestones presented with mild headaches (2–3 times per month), exercise intolerance, gastro‐intestinal dysfunction, and delayed physical development since the age of 9. She was then diagnosed with mitochondrial encephalopathy, as suggested by elevated serum lactate levels and a pathogenic variant (m.3243A>G) in the mitochondrial tRNA leucine 1 gene (mutational load, 55%). Co‐enzyme Q10, vitamin‐B, and levocarnitine were prescribed. At the age of 13 she had her first stroke‐like episode manifesting as right hemiparesis, muscle weakness, and right hemianopsia. Magnetic resonance imaging (MRI) examination revealed a lesion in the left posterior quadrant, mild cortical volume loss, and bilateral basal ganglia calcification (Figure 1). She also experienced focal unawareness seizures with left deviation of the head and eyes, which sometimes developed into bilateral tonic‐conic seizures. The seizures were initially controlled by oxcarbazepine.

**FIGURE 2:**
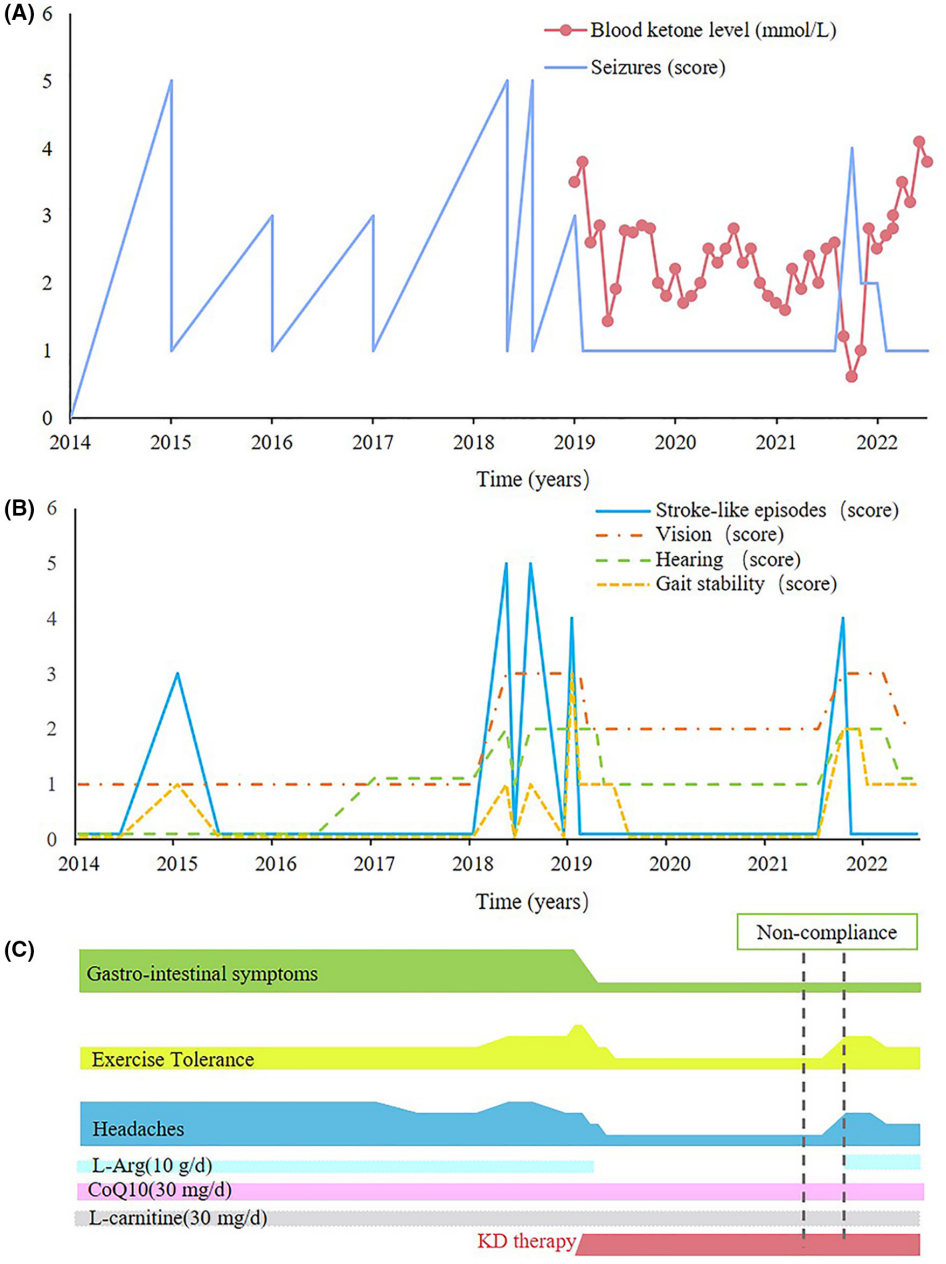
Changes in the symptom severity before and after KD therapy. The severity of the symptoms was assessed by the newcastle mitochondrial disease adult scale (NMDAS). (A) Seizures score scale shown as continuous light purple lines. The weekly blood ketone levels after KD treatment were averaged per month and shown as a continuous red solid dot at each time point. (B) Stroke‐like episodes score scale shown as continuous light blue lines. Vision score scale shown as continuous light orange lines. Hearing score scale shown as continuous light green lines. Gait stability score scale shown as continuous light yellow lines. (C) The KD therapy improved other symptoms independent on stroke‐like episodes including exercise intolerance, gastrointestinal function, and headaches in this MELAS‐patient. CoQ10, coenzyme; KD, ketogenic diet; MELAS, mitochondrial encephalopathy with lactic acidosis and stroke‐like episodes; Q10; L‐Arg, L‐arginine.

Since the age of 16 she was repeatedly hospitalized for uncontrolled seizures and psychotic symptoms despite being on a course of multiple anti‐seizure medications (Figure 2). She also had deteriorating hearing loss and paramenia. She then experienced three additional stroke‐like episodes involving the left occipitoparietal region and was referred to our hospital. On admission she showed severely impaired cognition, elevated serum lactate level (3.2 mmol/L at resting state), weakness of the right limbs (muscle strength rating, grade 3), right homonymous hemianopsia, and retractable epilepsies partials continua. We initiated KD at a 4:1 ratio to control the seizures. Three days later the seizures quickly reduced with a corresponding blood ketone level of 4.0 mmol/L. She was able to walk and discharged after 1 week. She continued KD therapy and anti‐seizure medications were gradually reduced. She remained seizure‐free on monotherapy of levetiracetam. Interestingly, she reported marked improvement in many aspects, including cerebral symptoms associated with stroke‐like episodes (vision, hearing, and gait stability) and the other symptoms independent of stroke‐like episodes (headaches, gastrointestinal function, and exercise intolerance). No new lesion was found on MRI examination and the electroencephalographic examinations showed an improvement in posterior background. She returned to school several months later. The diet ratio was gradually reduced to 2:1 at 9 months after KD initiation, and the blood ketone level (measured weekly) was maintained at around 2.0 mmol/L.

She remained free of seizures and stroke‐like episodes for 2.7 years until non‐compliance with the prescribed diet and a drop in blood ketone levels. Following this, she was hospitalized during a fifth stroke‐like episode that presented with convulsive status epilepticus, exacerbated visual/hearing function, sleep disturbance, and psychiatric symptoms. Strict KD therapy was restarted and she has remained seizure‐free to the present. The new lesions in the bilateral frontal lobes and the right parietal lobe resolved. After restarting strict KD treatment, prominent improvement was observed again in gait stability, visual function, and cognition. Some mild side effects were found during KD therapy, including hypertriglyceridemia and paramenia. She gained body weight and the body mass index was normalized (pre‐treatment, 17.1 kg/m^2^; post‐treatment, 18.5 kg/m^2^) due to improved gastrointestinal function.

## DISCUSSION

MELAS syndrome is a maternally inherited multisystemic disorder. Our patient had multiple symptoms including chronic headaches, lactic acidosis, exercise intolerance, drug‐resistant epilepsy, and stroke‐like episodes. On the prolonged KD therapy, the patient achieved seizure‐freedom for 2.7 years and a delayed relapse of stroke‐like episode. Additionally, many symptoms were prominently improved, including headaches, motor function, hearing and visual function, and gastrointestinal function. The stroke‐like episode recurred with diet incompliance, suggesting a sufficient and stable ketosis may be necessary to maintain the efficacy of KD in MELAS syndrome. Compared to a patient with MELAS syndrome who was seizure‐free for 1 year with KD therapy,^5^ our patient experienced improvement in several typical symptoms of MELAS, as well as a longer duration of seizure control. These evidences lend support to the long‐term use of ketogenic diet as a safe and effective method for treating MELAS syndrome.

In MELAS syndrome the dysfunctional mitochondria are unable to generate sufficient energy to meet the needs of multiple organs.^6^ KD contains high‐fat, low‐carbohydrate, and adequate‐protein, and it simulates a metabolic condition of fasting in the body and generates ketone bodies in the liver. KD therapy possibly exerts its positive effects via stimulating mitochondrial biogenesis, improving mitochondrial function, and decreasing oxidative stress.^3^ As a common mitochondrion disorder, MELAS syndrome is associated with multiple partial respiratory chain defects mainly involving complex I and/or IV deficiencies.^7^ One study found ketone bodies could restore complex I stability and activity, increase ATP synthesis, and reduce the NADH/NAD^+^ ratio.^8^ Glucose restriction can improve complex I assembly and oxidative phosphorylation function in a neuronal‐like model of MELAS syndrome.^7^ Ketone bodies may enhance NADH oxidation and markedly increase complex I‐driven mitochondrial respiration. In addition, ketone bodies increased the absolute amount of wild type mtDNA copy number in mutant cells without changing the mutant load.^9^ The multi‐facet beneficial effects of KD in our patient might be due to increased wild type mtDNA copy number thereby improving energy metabolism in the peripheral nerves, muscle, gastrointestinal tract, and brain.

Re‐introduction of the strict ketogenic diet only partially improved her symptoms, suggesting a progressive nature of MELAS syndrome. The length of time KD therapy should be maintained in MELAS syndrome requires further investigation. The treatment duration largely depends on an individual's tolerance and compliance to the diet. Further clinical studies are warranted to verify the efficacy of KD in MELAS syndrome.

## AUTHOR CONTRIBUTIONS

Fang He & Lingqi Ye: planned and monitored the KD therapy, analyzed the data, drafted the initial manuscript. Pu Miao: participated in patient's management, collected the history. Jiong Zhou &Yao Ding: participated in patient's management. Shuang Wang: conceptualized the study and revised the manuscript.

## FUNDING INFORMATION

This study was supported by the National Natural Science Foundation of China (No.81971207, 82,171,437, 81,971,208, 82,171,889) and funding from the China Association Against Epilepsy (No. CJ‐A‐2021‐11).

## CONFLICT OF INTEREST STATEMENT

The authors declare that the research was conducted in the absence of any commercial or financial relationships that could be construed as a potential conflict of interest.

## Data Availability

Not applicable.
